# Live Detection of Intracellular Chitin in Butterfly Wing Epithelial Cells In Vivo Using Fluorescent Brightener 28: Implications for the Development of Scales and Color Patterns

**DOI:** 10.3390/insects14090753

**Published:** 2023-09-08

**Authors:** Yugo Nakazato, Joji M. Otaki

**Affiliations:** The BCPH Unit of Molecular Physiology, Department of Chemistry, Biology and Marine Science, Faculty of Science, University of the Ryukyus, Okinawa 903-0213, Japan

**Keywords:** bioimaging, butterfly wing, chitin, cuticle, epithelial cell, fluorescent brightener 28, fluorescent probe, in vivo imaging

## Abstract

**Simple Summary:**

Chitin is the major component in the extracellular cuticle and plays multiple roles in insects. In butterflies, chitin contributes to the development of scales and color patterns. Here, we attempted to detect intracellular chitin in live cells with fluorescent brightener 28 (FB28), focusing on wing epithelial cells of a small lycaenid butterfly. We observed strongly FB28-positive cells in the cytosol, which may be specialized chitin-secreting cells. We detected FB28-positive hexagonal intracellular objects and their associated structures extending toward the apical end of the cell, which may be developing scale bases and shafts. We also observed FB28-positive fibrous intracellular structures extending toward the basal end. The present data are crucial to understanding the differentiation of the butterfly wing epithelium, including scale formation and color pattern determination. The use of FB28 in probing intracellular chitin in live cells may be applicable to other insect systems.

**Abstract:**

Chitin is the major component of the extracellular cuticle and plays multiple roles in insects. In butterflies, chitin builds wing scales for structural colors. Here, we show that intracellular chitin in live cells can be detected in vivo with fluorescent brightener 28 (FB28), focusing on wing epithelial cells of the small lycaenid butterfly *Zizeeria maha* immediately after pupation. A relatively small number of cells at the apical surface of the epithelium were strongly FB28-positive in the cytosol and seemed to have extensive ER-Golgi networks, which may be specialized chitin-secreting cells. Some cells had FB28-positive tadpole-tail-like or rod-like structures relative to the nucleus. We detected FB28-positive hexagonal intracellular objects and their associated structures extending toward the apical end of the cell, which may be developing scale bases and shafts. We also observed FB28-positive fibrous intracellular structures extending toward the basal end. Many cells were FB28-negative in the cytosol, which contained FB28-positive dots or discs. The present data are crucial to understanding the differentiation of the butterfly wing epithelium, including scale formation and color pattern determination. The use of FB28 in probing intracellular chitin in live cells may be applicable to other insect systems.

## 1. Introduction

Bioimaging has been a critical field of advanced technologies in biological sciences. Among others, the technological development of real-time confocal laser scanning microscopy (CLSM) has contributed to the advancement of molecular and cellular biology to reveal molecular and cellular dynamics in living cells [1,2]. In parallel with the advancement of optical hardware technology, chemically or biologically engineered fluorescent indicators have been developed [3,4,5], and they have played a pivotal role in bioimaging since the invention and application of calcium indicators in 1980 [6,7] and green fluorescent protein (GFP) in 1994 [8]. More fundamentally, organelle-specific fluorescent probes provide basic information on cellular and subcellular morphology [9,10,11,12]. Detection of specific intracellular structures depends greatly on the availability of specific fluorescent probes for such structures.

The exoskeleton (also called the integument) is a vital component of insects and other organisms. The exoskeleton is an extracellular cuticle structure secreted from epithelial (epidermal) cells [13,14,15,16,17]. The exoskeletal cuticle is stratified into the outermost envelope, the outer epicuticle, and the inner procuticle [13,14,15,16,17]. The procuticle can be divided into the outer exocuticle and the inner endocuticle, which contacts the apical side of the epithelial cells [13,14,15,16,17]. One of the major components of the procuticle is chitin [13,14,15,16,17]. Chitin is synthesized by the apical plasma membrane of epithelial cells [18] and is known to function as a vital molecule to produce mechanical rigor and morphological traits in insects [19,20,21,22,23]. Despite its importance, chitin detection methods are generally limited. An important fluorescent probe for chitin is fluorescent brightener 28 (FB28), also known under the names of calcofluor white and others [20,21,22,23]. Note, however, that FB28 also stains other polysaccharides such as cellulose [24,25,26,27] and that FB28 also stains the cytoplasm weakly and nuclei strongly when the plasma membrane is disrupted [27]. More recently, additional probes have been reported to detect cellulose and chitin [28,29]. In *Drosophila*, a genetic reporter for chitin called ChitVis-Tomato has been invented [30], but its application to nonmodel organisms has not yet been reported.

In butterflies, chitin is crucial in constructing wing scales together with F-actin [31,32,33]. Chitin thus directly contributes to the structural colors of butterfly scales [34,35,36,37,38]. In one study, chitin localization was probed with wheat germ agglutinin (WGA), which is one of the common stains for polysaccharides [31]. In another study, chitin was probed with a fusion protein containing a chitin-binding domain [32]. Recently, intracellular chitin was investigated using FB28 [39]. In these studies [31,32,39], only fixed tissues or nonliving specimens were studied. In a different study, FB28 was used to monitor extracellular chitin in living tissues in vivo in butterfly pupal wings [40]. However, because chitin has been recognized as an extracellular molecule synthesized at the apical plasma membrane, its intracellular localization in live cells in insects has not been well reported.

In the present study, we visualized intracellular chitin with FB28 in live cells in vivo in butterfly pupal wings. In insects, it can be safely assumed that FB28-positive signals are indications of chitin except nuclei; FB28 signals for chitin and nuclei should be distinguished by researchers. We used the pupal wings immediately after pupation when the majority of epithelial cells may still be considered common precursors of scale cells and socket cells [31,41]. At least some epithelial cells must actively secrete cuticle components, including chitin, to form the pupal cuticle. In addition, a very early stage of scale formation could be observed. We therefore examined whether there were epithelial cells containing intracellular chitin. Taking advantage of the fact that pupal wing tissues are located on the surface of the pupal body, forewing-lift operations have been performed to directly observe live wing epithelial tissues and cells in vivo since 2009 [42] and have been used for bioimaging [43,44,45,46,47,48,49] and for studying color pattern changes [40]. In this study, using this method, FB28 and other fluorescent probes were applied, and stained live cells and their intracellular structures were visualized in vivo.

## 2. Materials and Methods

### 2.1. Butterfly Rearing

We focused on butterfly pupal wing epithelial tissues as the system of choice. Here, we used the pale grass blue butterfly *Zizeeria maha* (Lepidoptera, Lycaenidae). We collected adult female butterflies in the Nishihara Campus of the University of the Ryukyus and obtained eggs from them on the host plant, the creeping wood sorrel *Oxalis corniculata*. Larvae hatched from these eggs were reared with the host plant leaves in plastic containers at ambient temperatures (approximately 26 °C). Eggs were also collected from laboratory-reared adult females.

### 2.2. Experimental Operations

We have previously reported how to visualize the wing epithelial tissue of the pale grass blue butterfly in vivo using a confocal microscope system [48,49]. FB28 and other fluorescent probes were topically applied to the wing epithelial tissue using the sandwich method as follows. Immediately after pupation, the left forewing was lifted under a stereomicroscope using forceps. A 4-μL droplet (containing fluorescent probes) was placed on the surface of the dorsal hindwing and sandwiched between the ventral forewing and the dorsal hindwing. After incubation for one or two hours, the surfaces of the wing tissue were washed with phosphate buffered saline (PBS). The surfaces of the ventral forewing and dorsal hindwing were placed on a thin glass plate and covered with a piece of plastic wrap to prevent water evaporation. The ventral forewing was subjected to fluorescence confocal microscopy. The operation was started within 20 min postpupation. Note that it is critical to perform this operation immediately after pupation (before pupal cuticle hardens) to expose epithelial surfaces and to load fluorescent probes efficiently.

### 2.3. Fluorescent Probes

FB28 was obtained from Sigma‒Aldrich (St. Louis, MO, USA), which was supplied as a 25% aqueous solution. We mostly used 4.4–8.1% aqueous solutions for the sandwich application, although higher concentrations were tested up to 13.8%. FB28 has many synonyms, such as Fluostain I, Calcofluor White LRP, ST, or M2R, and Tinopal LPW or UNPA-GX. For confocal imaging of epithelial cells, in addition to FB28, we used SYBR Green I (Life Technologies, Carlsbad, CA, USA) for nuclear staining and BODIPY FL C_5_-ceramide complexed to BSA (Thermo Fisher Scientific, Tokyo, Japan) for staining hydrophobic membranous structures such as the plasma membrane, endoplasmic reticulum (ER), Golgi apparatus, and vesicles. Thus, BODIPY FL C_5_-ceramide is useful to identify the cellular shape and intracellular development of the synthesis machinery for chitin or other substances. In addition, we used LysoTracker Red (Thermo Fisher Scientific) for lysosomal staining and MitoRed (Dojindo Molecular Technologies, Rockville, MD, USA) for mitochondrial staining. These red dyes were used to highlight cytoplasmic identity. Fluorescent probes (excluding FB28) were diluted with dimethyl sulfoxide (DMSO). The final concentrations for the sandwich application were as follows: BODIPY FL C_5_-ceramide (38.0–50.0 μM), MitoRed (10.7 μM), and LysoTracker Red (5.5 μM). The original SYBR Green I solution supplied by the manufacturer was diluted 3.3–2.2 times to the final concentration before use.

### 2.4. Confocal Imaging

For confocal microscopy of the wing epithelium of the pale grass blue butterfly, we followed a previous study [48]. Briefly, we employed a Nikon A1^+^ ECLIPSE Ti confocal microscope system (Tokyo, Japan). Confocal images were acquired to optically cut horizontal serial sections and processed for vertical cross-sectional reconstitution images and three-dimensional reconstruction images using NIS-Elements AR 4.20.00 64-bit (Nikon, Tokyo, Japan). Excitation wavelengths by solid lasers were 405 nm, 488 nm, and 561 nm, for which filtered emission wavelengths were 425–475 nm, 500–550 nm, and 570–620 nm, respectively. Two kinds of objective lenses (20× and 100×) were used. The software’s zoom functions (20 × 4, 20 × 5, and 100 × 2) were often employed. From the surface of the wing to deeper levels, horizontal serial sections were obtained with 0.4–0.5 μm steps. Under these conditions, no autofluorescence was detected in this study.

## 3. Results

### 3.1. FB28 and SYBR Green I: Proximal Regions

**Figure 1:**
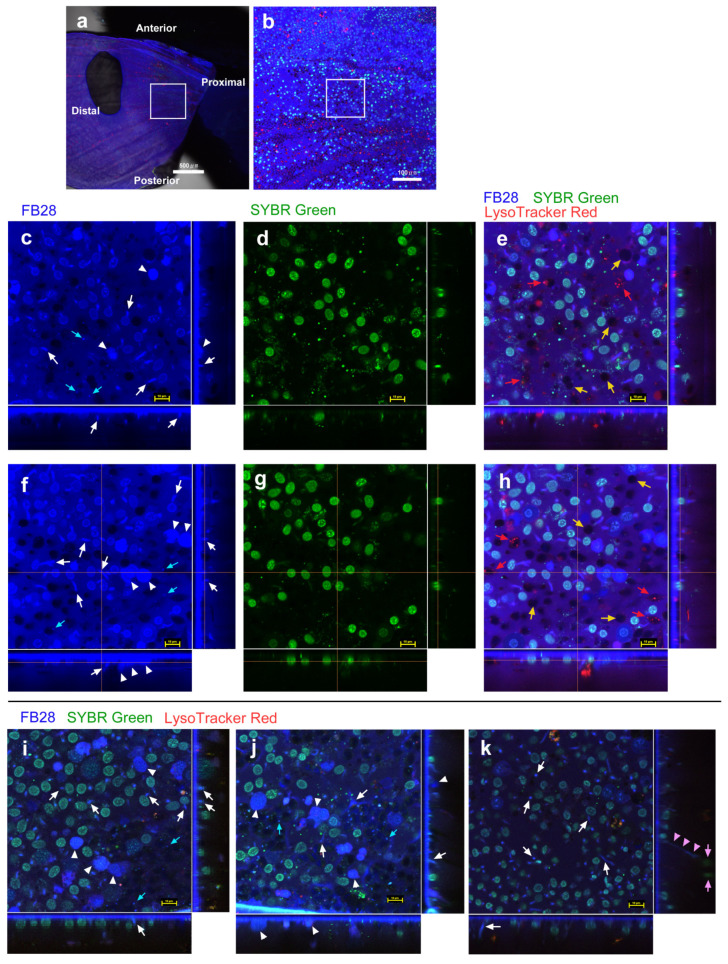
FB28 staining (blue) of pupal wing epithelial tissues. Tissues were also stained with SYBR Green I for nuclei (green) and LysoTracker Red for lysosomes (red). Each main panel is a horizontal optical section, which is accompanied by two minor panels of vertical cross sections at the right side and at the bottom (excluding (**a**,**b**)). In these vertical section panels, the apical extracellular site is shown as a blue continuous layer of FB28. White arrows indicate tadpole-tail-like structures. White arrowheads indicate strongly FB28-positive cells located at the apical surface within approximately 5–10 μm in depth. Red arrows indicate LysoTracker Red signals. Yellow arrows indicate blank circular areas (FB28-negative cells). Cyan arrows indicate FB28-positive dots or discs. Pink arrowheads indicate a downward fibrous structure. Pink arrows indicate SYBR-positive nuclei located at a depth of approximately 30–40 μm. FB28 concentration: 8.1%. (**a**) Ventral forewing. A squared proximal region is enlarged in (**b**). Scale bar: 500 μm. (**b**) Magnification of (**a**). A squared region is a wing compartment enlarged in (**c**–**e**). Scale bar: 100 μm. (**c**–**e**) Magnification of (**b**). These are a set of three images (FB28, SYBR Green I, and a merge of FB28, SYBR Green I, and LysoTracker Red) in the same visual field from an individual. (**f**–**h**) Another set of three images in the same visual field from another individual. Scale bars: 10 μm. (**i**–**k**) Images from three additional individuals. Each panel is a merged image of the three fluorescent probes (FB28, SYBR Green I, and LysoTracker Red). Scale bars: 10 μm.

We first used FB28 together with SYBR Green I and LysoTracker Red (*n* = 19 with 20× objective lens; *n* = 3 with 20× and 100× objective lenses) to distinguish FB28 signals for chitin and nuclei. FB28 stained the entire ventral forewing (Figure 1a). We first observed a proximal (basal–postbasal) region of the forewing (Figure 1b). We first noted that FB28 heavily stained the extracellular site of the apical surface of the epithelium because of extracellular chitin, producing a continuous blue layer in vertical sections (Figure 1c–k). As expected, FB28 weakly stained nuclei (Figure 1c), and SYBR Green I clearly stained nuclei (Figure 1d), which was confirmed by colocalization of FB28 and SYBR signals (Figure 1e). It appeared that FB28 nuclear staining was weaker than SYBR nuclear staining. It was not difficult to identify nuclei because they were round or oval in shape, were approximately 5–10 μm in diameter, and were located close to the apical surface (approximately within 5–10 μm in depth). Indeed, FB28 nuclear staining was observed only when using high concentrations (8.1% or more), as FB28-positive nuclei were scarce in subsequent figures using lower concentrations. Therefore, nuclear staining with FB28 did not pose any practical problems in this study.

Aside from nuclei, FB28 also stained the cytosol and intracellular structures in the epithelium of different cell populations. First, there were FB28-positive cells, the cytosol of which was strongly stained (Figure 1c). These strongly FB28-positive cells were not numerous here. In these cells, probably because of this strong FB28 staining, their nuclei were not stained well with SYBR Green I, but the nuclei were still visible in some of these cells, confirming that they were cellular entities. These cells seemed to be surface bound; their main body was located approximately within 5–10 μm in depth. Second, there were FB28-positive structures that seemed to be associated with the adjacent SYBR-positive nucleus, similar to a tadpole tail (Figure 1c). Some of these FB28-positive structures were also weakly positive for SYBR Green I (Figure 1d,e). These tadpole-tail-like structures were not always observed in individual samples, but instead other related FB28-positive structures were observed as frequently; they may be the early stage of developing scales (see Section 3.2). Third, there were numerous FB28-positive dots or discs in cells that were weakly FB28-positive and FB28-negative in their cytosol. Fourth, the cytosol of many epithelial cells appeared to be weakly stained (Figure 1c,e). This weak staining may be considered background noise but may define a group of cells that were lightly stained with FB28, considering that there were many cells completely negative for FB28. Indeed, FB28-negative cells were observed as blank circular areas, which were often associated with LysoTracker Red signals (Figure 1e). These blank areas must also contain nuclei [44,46,48]. Thus, some nuclei did not seem to be stained at all, and this staining difference may be explained by cell-dependent permeability.

These observations were confirmed in additional individuals from similar regions (Figure 1f–k). Notably, many strongly FB28-positive cells were observed as a twin in these images, and some of these cells seemed to be slightly larger than other surrounding cells. They were also surface bound, as were other surrounding cells (within approximately 5–10 μm in depth). Nucleus-associated structures (including the tadpole-tail-like structures) varied in shape (Figure 1i–k), and some of them were rod-like and vertically oriented (Figure 1i–k). In some cases, FB28-positive dots were observed together near the nucleus-associated structures. We occasionally observed fibrous structures extending to a deeper level (Figure 1k), probably connecting to deep cells or to the basal membrane. We could not observe this fibrous structure in every cell. This may be either because of technical reasons (i.e., difficulty in staining and observing deep and thin structures) or because of the rarity of this structure.

### 3.2. FB28 and BODIPY: Distal Regions

**Figure 2:**
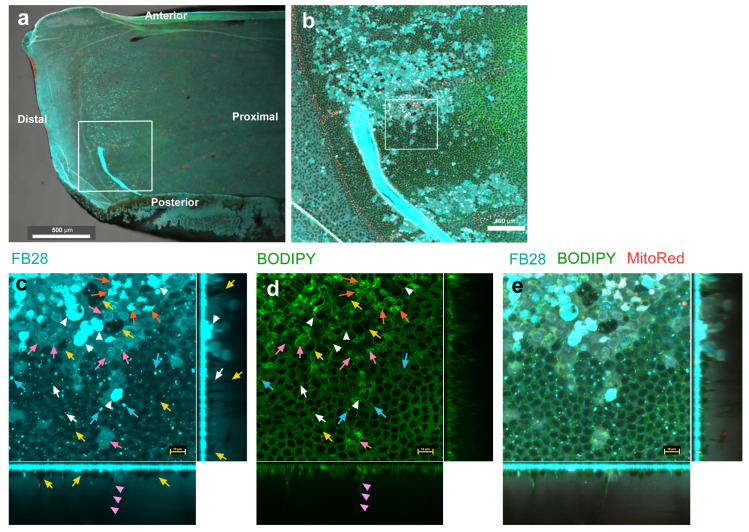
FB28 staining (cyan) of pupal wing epithelial tissues. Tissues were also stained with BODIPY FL C_5_-ceramide for membranous structures (green) and MitoRed for mitochondria (red). Each main panel is a horizontal optical section, which is accompanied by two minor panels of vertical cross sections at the right side and at the bottom (excluding (**a**,**b**)). White arrowheads indicate relatively large FB28-positive cells. White arrows indicate FB28-positive dots. Yellow arrows indicate blank circular areas (FB28-negative cells). Pink arrows indicate weakly FB28-positive cells with weakly stained nuclei. Orange arrows indicate relatively large nonnuclear FB28-positive areas, likely part of the cytoplasm. Pink arrowheads indicate FB28-positive fibrous structures. Cyan arrows indicate FB28-positive hexagonal objects. FB28 concentration: 7.4%. (**a**) Ventral forewing. A squared distal region is enlarged in (**b**). Scale bar: 500 μm. (**b**) Magnification of (**a**). A squared region is enlarged in (**c**–**e**). Scale bar: 100 μm. (**c**–**e**) A set of three images (FB28, BODIPY, and a merge of FB28, BODIPY, and MitoRed) in the same visual field from an individual. Note that in the upper left side of the major image, most cells are FB28 positive in their cytosol, which is overwrapped with higher intracellular BODIPY signals. Scale bars: 10 μm.

**Figure 3:**
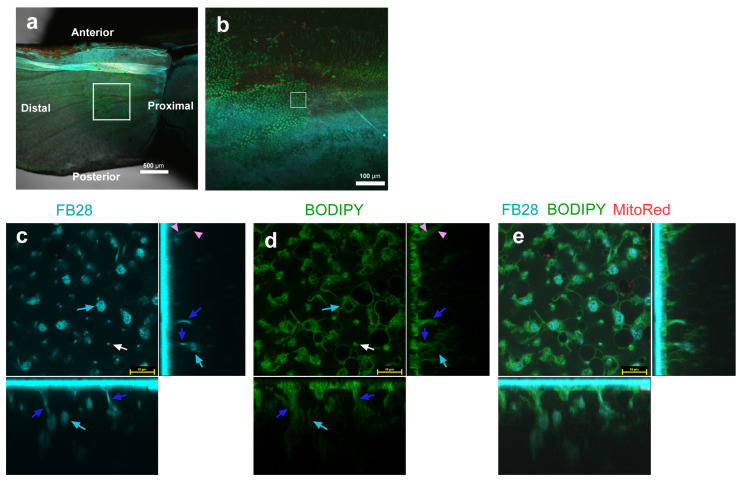
FB28 staining (cyan) of pupal wing epithelial tissues. Tissues were also stained with BODIPY FL C_5_-ceramide for membranous structures (green) and MitoRed for mitochondria (red). Each main panel is a horizontal optical section, which is accompanied by two minor panels of vertical cross sections at the right side and at the bottom (excluding (**a**,**b**)). White arrows indicate FB28-positive dots. Pink arrowheads indicate FB28-positive fibrous structures. Cyan arrows indicate FB28-positive hexagonal objects. Blue arrows indicate rod-like structures that may be connected with hexagonal objects. FB28 concentration: 4.4%. (**a**) Ventral forewing. A squared middle region is enlarged in (**b**). Scale bar: 500 μm. (**b**) Magnification of (**a**). A squared region is enlarged in (**c**–**e**). Scale bar: 100 μm. (**c**–**e**) A set of three images (FB28, BODIPY, and a merge of FB28, BODIPY, and MitoRed) in the same visual field from an individual. This individual is different from that of Figure 2. Scale bars: 10 μm.

**Figure 4:**
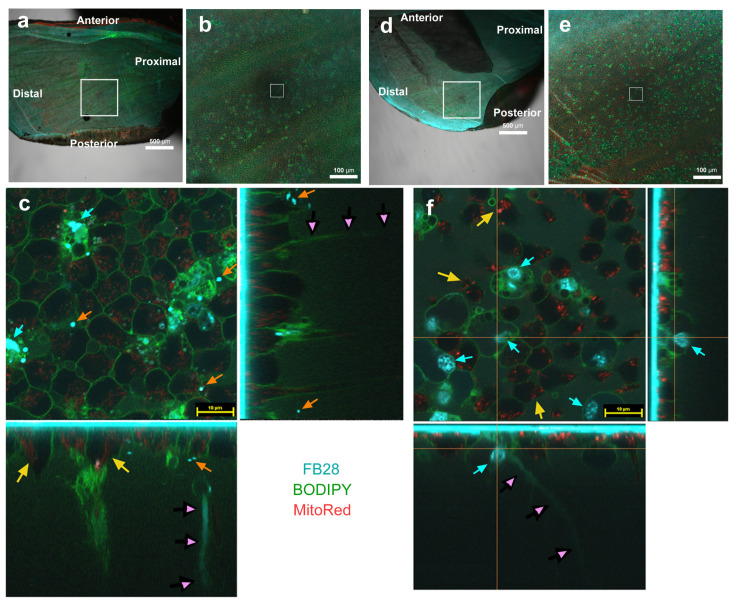
FB28 staining (cyan) of pupal wing epithelial tissues. Tissues were also stained with BODIPY FL C_5_-ceramide for membranous structures (green) and MitoRed for mitochondria (red). Each main panel is a horizontal optical section, which is accompanied by two minor panels of vertical cross sections at the right side and at the bottom (excluding (**a**,**b**)). FB28 concentration: 4.4%. (**a**) Ventral forewing. A squared middle region is enlarged in (**b**). Scale bar: 500 μm. (**b**) Magnification of (**a**). Scale bar: 100 μm. (**c**–**f**) Each panel is a merged image of the three fluorescent probes. In these individuals, relatively large FB28-positive intracellular hexagonal objects are observed (cyan arrows). There are FB28-positive dots or discs (orange arrows). FB28-positive fibers extending downward to the basal end are observed (pink arrowheads). FB28-negative cells with MitoRed-positive dots (mitochondria) are seen (yellow arrows). Scale bars: 10 μm.

To examine whether FB28-positive structures are intracellular and whether epithelial cells have ER-Golgi networks possibly for synthesizing and secreting chitin, we next used BODIPY FL C_5_-ceramide for staining hydrophobic membranous structures together with LysoTracker Red (*n* = 7 with 20× objective lens; *n* = 3 with 20× and 100× objective lenses), MitoRed (*n* = 12 with 20× objective lens; *n* = 11 with 20× and 100× objective lenses), or MitoRed and SYBR Green I (*n* = 8 with 20× objective lens) (Figure 2, Figure 3 and Figure 4). Here, we observed a distal (marginal–submarginal) region of the ventral forewing (Figure 2a). FB28-positive cells were abundant near the distal ends of wing veins and were relatively scarce along the midline of wing compartments (Figure 2b).

As expected from Figure 1, FB28 strongly stained relatively large cells of approximately 10 μm in diameter (Figure 2c). Their cellular outlines were confirmed with a BODIPY-positive plasma membrane (Figure 2d,e). These strongly FB28-positive cells had a BODIPY-negative region inside a cell, probably corresponding to the nucleus, and a relatively complex and high-density BODIPY-positive region inside a cell, suggesting an active ER-Golgi network. In addition, there were cells in which the cytosol was weakly FB28-positive (Figure 2c). The nuclei of these cells were also stained with FB28. In some of these cells, parts of the cytosol seemed to be strongly stained with FB28 (Figure 2c). These FB28-positive cytosolic areas overlapped extensively with BODIPY signals (Figure 2c–e), again suggesting an extensive ER-Golgi network probably for ongoing chitin production. To be sure, in the vertical sections, the apical epithelial surface showed a BODIPY-positive layer, likely covering all cells in the epithelium (Figure 2d). Thus, most, if not all, cell types may have active ER-Golgi networks near the apical membrane. However, more BODIPY signals were found in FB28-positive cells than in FB28-negative cells.

There were many cells that were FB28-negative in their cytosol (Figure 2c). They contained FB28-positive dots or discs, and these dots or discs were numerous in the epithelium (Figure 2c,e). The size of the dots varied, and some may be called discs. Larger ones were approximately 5 μm or less in size and may be called FB28-positive objects instead of the tadpole-tail-like or rod-like structures found in Figure 1 (Figure 2c). In contrast to proximal regions (Figure 1), we did not observe clear tadpole-tail-like or rod-like structures in distal regions (Figure 2). In addition, as shown in Figure 1, we observed FB28-positive fibrous structures extending deep toward the basal membrane or deep cells (Figure 2c,e), which were also BODIPY positive (Figure 2d,e), suggesting that these fibrous structures were intracellular.

### 3.3. FB28 and BODIPY: Middle Regions

We then observed the middle (postbasal–discal) regions of the ventral forewing (Figure 3a,b) using BODIPY as described above. To further explore the FB28-positive objects in FB28-negative cells found above, we obtained higher magnification images. Strongly FB28-positive objects with various irregular shapes (not round or oval) were clearly detected; they were approximately 5 μm in size in horizontal sections (Figure 3a–e). Some of them seemed to be hexagonal in the horizontal sections and oval in the vertical sections. They were surrounded by BODIPY signals, suggesting that they were intracellular. Horizontally speaking, these objects were located at a peripheral site of a cell, like an appendage. Vertically speaking, they were located at a deeper level (approximately 5–20 μm in depth) than the level of nuclei. These hexagonal objects appeared to be connected with rod-like structures extending upward toward the apical side (Figure 3c–e), which were also surrounded by BODIPY signals, suggesting that the rod-like structures were also intracellular. These rod-like structures are probably essentially the same object as the tadpole-tail-like and rod-like structures found in Figure 1. FB28-positive dots were also overwrapped with BODIPY, suggesting that they were also surrounded by membranous structures.

Additionally, in different individuals with higher magnification of a middle (discal) region (Figure 4a,b), as well as a more distal (submarginal tornus) region (Figure 4d,e) of the ventral forewing, we clearly observed FB28-positive objects (Figure 4c,f). They were irregular in shape (not round or oval) (Figure 4c), but some of them showed hexagonal shapes in the horizontal sections and oval shapes in the vertical sections (Figure 4c,f). They were extensively associated with BODIPY signals. These features were the same as those in Figure 2 and Figure 3. However, unlike the individuals shown in Figure 3, the FB28-positive hexagonal objects were clearly located within a part of a larger cell adjacent to the plasma membrane (Figure 4c,f). In the horizontal plane, MitoRed signals were confined within blank circular areas, similar to LysoTracker signals (Figure 4c,f). In the vertical plane, MitoRed signals were located mostly at the apical surface (Figure 4c,f). Because MitoRed signals were immediately associated with nuclei in previous studies [43,44,45,46], the blank circular areas must be mostly occupied by nuclei. In contrast, the FB28-positive objects were not associated with mitochondrial signals, but this may simply be because these cells were highly positive for BODIPY, which might have interfered with MitoRed staining. Numerous FB28-positive dots or discs were also observed (Figure 4c). We additionally observed FB28-positive fine fibers extending downward to the basal side (Figure 4c,f).

### 3.4. Three-Dimensional Images

**Figure 5:**
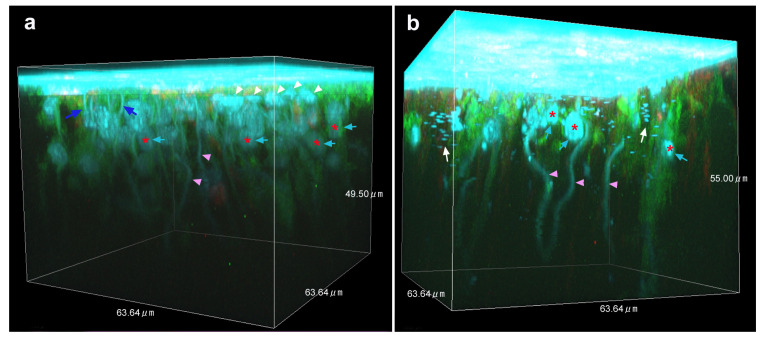
Three-dimensional reconstruction images of optical sections stained with FB28 (cyan), BODIPY FL C_5_-ceramide (green), and MitoRed (red). See also movies for these panels (Supplementary Materials). At the apical surface, there are cells that are highly positive for FB28 in the cytosol (white arrowheads). FB28-positive objects are indicated by cyan arrows together with red asterisks. There are FB28-positive rod-like structures extending upward (blue arrows). There are also FB28-positive fibrous structures extending downward (pink arrowheads). These structures are associated with BODIPY signals. FB28-positive dots or discs are observed (white arrows). FB28 concentration: 4.4%. (**a**) Cuboid from an individual. (**b**) Cuboid from another individual.

We produced three-dimensional reconstruction images of epithelial staining with FB28, BODIPY, and MitoRed in the middle regions where many FB28-positive objects were observed (*n* = 3) (Figure 5; Supplementary Materials). We confirmed the findings in the previous sections: surface-bound FB28-positive cells within approximately 5–10 μm in depth from the surface, FB28-positive objects at a depth of approximately 5–20 μm from the surface, rod-like structures, FB28-positive dots or discs, and downward fibrous structures (Figure 5; Supplementary Materials). These FB28 signals were associated with intracellular BODIPY signals. The downward fibrous structures may not be associated with other structures and seemed to extend from the apical to the basal ends, but this was uncertain in these images.

## 4. Discussion

The present study revealed intracellular chitin in living insects using FB28 and the butterfly wing system. FB28 has been used to probe extracellular chitin and cellulose in histological studies in fixed tissues [24,25,26,27,39], and the current study presented a case where a well-known fluorescent probe revealed new intracellular structures when applied in live cells. From a technical viewpoint, we assumed that FB28 signals indicate the presence of chitin. Although FB28 may also bind to other polysaccharides, this assumption is likely reasonable, considering that most structural polysaccharides in insects are chitin. An additional technical concern was that, likely because DNA has a deoxyribose moiety in the backbone, nuclei were occasionally weakly positive for FB28 when used at high concentrations (e.g., 8.1%). Nuclear staining with FB28 has been reported, which can be applied for a cell viability assay using low concentrations (0.01–0.03%) [27]. Overall, the present study used much higher concentrations (4.4–8.1%), although it may be diluted in situ. Despite this unfavorable staining, SYBR Green I appeared to have a higher affinity for DNA, and thus, it was relatively straightforward to distinguish chitin from nucleic acids. Indeed, lower concentrations of FB28 (e.g., 4.4%) in our system mostly eliminated nuclear staining with FB28.

Another technical viewpoint, related to the point above, is the cellular permeability of FB28. It was fortunate that FB28 crossed the cell membrane easily at the concentrations used in this study. No chemical modifications are necessary for cellular penetration before use, although the extracellular layer was stained much more strongly than the intracellular structures. Furthermore, FB28 is easy to handle because it is used as an aqueous solution, and it is available at low cost. Other dyes for polysaccharides, such as Solophenyl Flavine 7GFE 500 and Pontamine Fast Scarlet 4B, may also be applicable [28], but FB28 is more economical. On the other hand, the use of a genetic reporter for *Drosophila*, ChtVis-Tomato [30], may be challenging in butterflies and other insects. Therefore, FB28 is a reasonable choice to probe the intracellular structures and dynamics of chitin in live insects.

**Figure 6:**
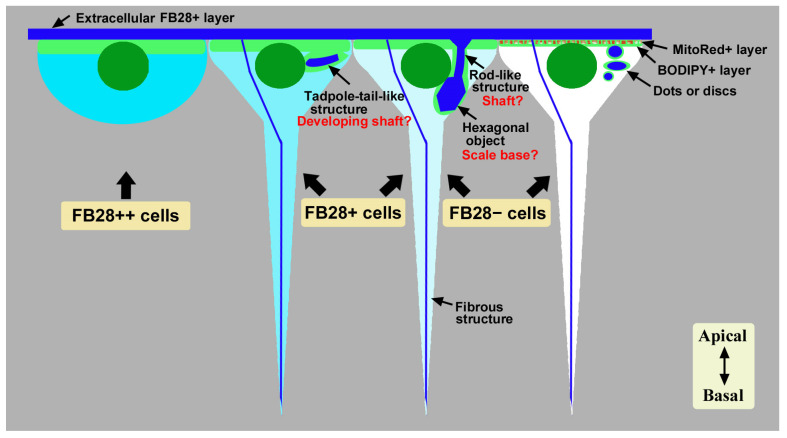
Summary of cytosolic FB28-positive cells (cyan) and FB28-positive intracellular structures (blue) detected in pupal wing epithelial cells. Strongly FB28-positive (++) cells may be chitin-secreting cells. Weakly FB28-positive (+) cells may contain tadpole-tail-like structures and other structures, such as hexagonal objects that may have upward rod-like structures. Cytosolic FB28-negative (−) cells may contain FB28-positive dots or discs. There is a single fibrous structure per cell, but whether all cells possess this structure is uncertain. In this figure, this fibrous structure is not associated with other intracellular structures, but the relationship between the fibrous structure and other structures is uncertain in the present study. The thickness of the BODIPY-positive (+) layer varies in this figure. The MitoRed-positive (+) layer is superimposed on the BODIPY-positive (+) layer. A hexagonal object should be oval in vertical sections, but here it is depicted hexagonal just for a symbolic icon.

The main results of the present study are summarized in Figure 6. In the pupal wing system investigated here, the apical surface of the epithelial tissue was covered with cuticle containing chitin. Below this FB28-positive extracellular layer, there was an epithelial cell layer in which a few cell types were notable in terms of their cytosolic FB28 staining: strongly FB28-positive cells, weakly FB28-positive cells, and FB28-negative cells. The first cell type was relatively large and surface bound. They may be without long basal processes, although this is uncertain. These strongly FB28-positive cells were more abundant in the distal region along the wing veins than in the proximal region. We speculate that they are chitin-synthesizing and/or chitin-secreting cells because they seem to have more extensive ER-Golgi networks than other cells at the apical surface. These potential chitin-secreting cells may be specialized (or “professional”) for secretion. Because the extracellular cuticle is critical for color pattern determination in butterflies [40,50], these potential chitin-secreting cells may play an important role in determining the color of scales produced by scale cells. However, other cell types (i.e., scale cells and socket cells) may also secrete chitin for the extracellular cuticle because all cell types seem to have ER-Golgi networks at the apical surface to various degrees. We cannot exclude the possibility that strongly FB28-positive cells are precursor cells for scale and/or socket cells. FB28-negative cells also constitute an epithelial layer, and they are more numerous than FB28-positive cells. Interestingly, they had intracellular FB28-positive dots or discs.

Weakly FB28-positive cells and FB28-negative cells in their cytosol contained tadpole-tail-like structures, rod-like structures, and/or relatively large FB28-positive hexagonal objects. Tadpole-tail-like structures and rod-like structures may possibly be developing scale shafts, and the hexagonal objects may be scale bases. That is, these cells may already function as scale cells in this lycaenid butterfly, although the epithelium at this time point may be believed to be composed of common precursor cells for scale and socket cells in nymphalid butterflies [31,41]. Along this line of discussion, the FB28-positive dots or discs in FB28-negative cells may serve as a core for a future scale shaft or base. Alternatively, the dots or discs may simply be chitin being secreted for extracellular cuticle. In *Drosophila*, intracellular chitin vesicles and punctae have been observed in a mutant background overexpressing the *kkv* gene, which encodes a chitin synthase [51]. It is likely that high-level chitin production by a chitin synthase results in intracellular chitin vesicles and punctae [51]. These chitin vesicles and punctae in *Drosophila* are probably similar to the FB28-positive dots or discs detected in the present study, suggesting high-level chitin production in butterfly epithelial cells. Including the dots or discs, all the structures detected in the present study seem to be intracellular at the developmental time of observations.

As mentioned above, we speculate that tadpole-tail-like structures are developing scale shafts. It seems that this structure is arranged nearly horizontally (not vertically). Horizontal structures should be vertically arranged later, which will become rod-like structures. Alternatively, this structure may simply serve as a core for very early scale development. Interestingly, this structure seems to be positioned at a defined site with respect to a nucleus and perpendicular to a nucleus. This fact may suggest that chitin-based scales are produced from the centrosome (microtube organizing center). It should be noted that the centrosome plays an important role in cilia generation in sperm [52,53,54,55]. On the other hand, we did not observe any planar cell polarity (PCP) of the tadpole-tail-like structures or any other structures in the epithelium at the time of observations in this study, but long-term recordings would detect the emergence of PCP. In *Drosophila*, many PCP genes have been identified [56,57,58,59], and the Dusky-like (Dkl) protein is required for chitin deposition to attach the apical cell membrane to chitin [60,61]. Similar genes may operate in butterflies.

The hexagonal objects appeared to be more prominent in the middle regions of the wing. Tadpole-tail-like or rod-like structures were observed in the proximal regions, as well as in the middle regions to a lesser extent. In the distal regions, these structures were less clear. These spatial differences are consistent with our notion that the development of the wing is probably the fastest in the middle regions where the discal spot is located. Whether early development of hexagonal objects is dependent on tadpole-tail-like or rod-like structures is uncertain, but we speculate that early development of the scale base is critical for subsequent scale development. The importance of the scale base at the early stage of cellular differentiation may be understood from the viewpoint of construction engineering; an entire scale is much larger than the base structures, which may not be realized without rigid bases. We do not know from the present study how socket cells come into play, but socket differentiation is probably executed later to encapsulate a scale shaft at the very apical site. Long-term in vivo observations may solve this issue in the future.

In addition, there was a relatively thin fibrous structure toward the basal end. The downward fibrous structure may be present as early as when the basal processes are established. A butterfly wing epithelial cell has a long basal process [44,48], and the tip of a basal process seems to form a “synapse” with deep cells. Thus, the fibrous structure may function as a cytoskeleton in the basal process to anchor it to a basal cell or basal membrane. In that case, chitin may function together with other conventional cytoskeleton components, such as actin filaments, microtubules, and intermediate filaments. Indeed, chitin fibers are aligned along with actin during scale development [31]. Only a single thread of the fibrous structure was noted per cell in the present study, and a thread may be composed of numerous fine chitin fibers. Alternatively, these fibrous structures may eventually differentiate into chitin-based bundle structures that connect the dorsal and ventral wings [62].

The long basal processes of epithelial cells are vertically oriented and relatively large cellular structures [44,48], in which we detected FB28-positive fibrous structures. On the other hand, we have also previously reported [44,48] that epithelial cells in butterfly wings have epidermal feet (epithelial feet) that were described in the 1980s [63,64,65,66,67] or cytoneme-like structures that were described more recently [68,69,70,71,72,73]. They are horizontally oriented and may function in cellular communications during development [63,64,65,66,67,68,69,70,71,72,73]. The present study did not detect any FB28-positive signals in these fine structures.

It has been reported that the precursor cell divides into two daughter cells, approximately 24 h postpupation [31], differentiating into a pair of scale and socket cells. Thus, it is surprising that intracellular chitin structures (possible scale shafts and bases) were found immediately after pupation in this lycaenid butterfly. However, in another study [46], cell divisions were observed just 1 h postpupation, and an increase in nuclear volume was observed 10 h postpupation. In fact, observational results somewhat varied from individual to individual in the present study. We think that there are slightly different developmental stages, even though our experimental protocol was standardized. Likely, the developmental time course with respect to pupation may vary among species, sexes, and regions of the wing tissue. In fact, we observed no FB28-positive intracellular structures (i.e., tadpole-tail-like structures, hexagonal structures, rod-like structures, fibrous structures, and dots or discs) immediately after pupation in a nymphalid species, *Junonia orithya* (unpublished data), although we have not yet examined the wing tissue of this species rigorously. Further studies are necessary to resolve relationships among the FB28-positive structures detected in this study.

Growing scales have been observed at time points much later in this species: 45 h postpupation [49]. In other species, growing scales have been observed 20 h [44] and 36 h [31] postpupation, and the detailed time course of scale development has been studied [31,32]. The present study is likely the first observation of the very early stages of scale development immediately after pupation. In other words, FB28 staining may be useful for monitoring growing scales from the very beginning of their growth. It should be noted that intracellular chitin production patterns may influence scale nanostructures for structural colors [34,35,36,37,38] and that scale growth real-time imaging may be fruitful in biological and materials sciences [74].

## 5. Conclusions

This study exemplified the usefulness of FB28 to study intracellular chitin-based structures in real-time imaging in live cells in vivo. Although chitin is known to be an extracellular molecule, intracellular chitin has not been well studied in live cells. In this sense, the present study provides researchers with a new opportunity to study intracellular chitin in insects. Here, possible chitin-secreting cells and developing scale shafts and bases were found together with downward fibrous structures in butterfly wing epithelial cells of *Z. maha* (Lepidoptera: Lycaenidae). These intracellular chitin-based structures may be important in the construction of scales (including their nanostructures), bundles (for dorsal and ventral surfaces), and color patterns in butterfly wings.

## Data Availability

All data relevant to the results and conclusions are included in this paper.

## Supplementary Materials

The following supporting information can be downloaded at https://zenodo.org/record/8084668 (accessed on 22 August 2023) as AVI files entitled “Three-dimensional reconstitution for Figure 4a” and “Three-dimensional reconstitution for Figure 4b” (https://doi.org/10.5281/zenodo.8084668 accessed on 22 August 2023).
